# Pituitary-Derived Circular RNAs Expression and Regulatory Network Prediction During the Onset of Puberty in Landrace × Yorkshire Crossbred Pigs

**DOI:** 10.3389/fgene.2020.00135

**Published:** 2020-02-28

**Authors:** Zitao Chen, Xiangchun Pan, Yaru Kong, Yao Jiang, Yuyi Zhong, Hao Zhang, Zhe Zhang, Xiaolong Yuan, Jiaqi Li

**Affiliations:** National Engineering Research Centre for Breeding Swine Industry, Guangdong Provincial Key Laboratory of Agro-Animal Genomics and Molecular Breeding, College of Animal Science, South China Agricultural University, Guangzhou, China

**Keywords:** pituitary, crossbred pig, puberty, circRNAs, miRNA sponges

## Abstract

Being the center of the hypothalamus-pituitary-ovary (HPO) axis, the pituitary plays a key role in the onset of puberty. Recent studies show that circular RNAs (circRNAs) can perform as miRNA sponges to regulate development in animals. However, the function of pituitary-derived circRNAs in first estrus remains unclear in pigs. In this study, we performed a genome-wide identification and characterization of circRNAs using pituitaries from Landrace × Yorkshire crossbred pigs at three stages: pre-, in-, and post-puberty, to describe such pituitary-derived circRNAs in pigs. A total of 5148 circRNAs were found in the gilts' pituitaries, averaging 18 682 bp in genomic distance, which consisted of approximately 91% exonic, 6% intergenic, and 3% intronic circRNAs. Furthermore, 158 novel circRNAs were identified for the first time and classified as putative pituitary-specific circRNAs. Their expression levels during the onset of puberty, significantly exceeded those of the other circRNAs, and the parental genes of these putative pituitary-specific circRNAs were enriched in “ssc04917: prolactin signaling pathway,” “ssc04080: neuroactive ligand-receptor interaction,” and “ssc04728: dopaminergic synapse” pathways, all of which were consistent with pituitary functioning. Additionally, 17 differentially regulated circRNAs were found and investigated for their potential interaction with miRNAs, along with genes, by constructing a circRNA-targeted miRNA-gene network. Taken together, these results provide new insight into the circRNA-mediated timing of puberty in gilts at the pituitary level.

## Introduction

Puberty can usually be defined as the first estrus of gilts, and the initiation of puberty implies the acquired capacity for sexual reproduction in pigs (Nonneman et al., 2016). An early onset of puberty can shorten the generation interval of livestock, and further accelerate the genetic breeding process (Rosales Nieto et al., 2014; Luo et al., 2017). Yet, surprisingly little is known about the molecular regulation of puberty's timing in pigs. Previous research has uncovered endocrinological differences across pubertal onset mainly driven by the hypothalamus-pituitary-ovary (HPO) axis (Angold et al., 1999; Blakemore et al., 2010). As the center bridging the hypothalamus and ovary in the HPO axis, the pituitary is an extremely important mediator for controlling the synthesis of hormones. During estrous cycling, an increase in the pulsatile release of gonadotropin-releasing hormone (GnRH) from the hypothalamus elicits an increased release of luteinizing hormone (LH) and follicle-stimulating hormone (FSH) from the pituitary (Root, 1980; Coe et al., 1981). Additionally, gonadotropin hormones released from the pituitary have been shown to be directly related to animals' reproductive associated traits (Barb et al., 2012).

Circular RNAs (circRNAs) are a novel type of circular RNA molecules lacking 5′–3′ polarities and polyadenylated tails (Chen and Yang, 2015), making them more structurally stable than linear RNAs (Qu et al., 2015). Most circRNAs consist of multiple exons, as well as introns of protein-coding genes, and are conserved among different animal species (Szabo and Salzman, 2016). With the advances made in next-generation sequencing technology, much research on circRNAs has been carried out using high-throughput RNA sequencing (RNA-seq). In pigs, recent work has demonstrated circRNAs' involvement in various organismal processes. For example, through a comprehensive analysis of porcine cardiac and skeletal muscles, Chen et al. (2018) showed that circRNAs contribute to differences in aging. Moreover, circRNAs were defined as a new biomarker in metabolism-related diseases based on a study of circRNAs occurring in the subcutaneous adipose tissues of two pig breeds (Li et al., 2018). For their role in estrus, Li et al. (2018) investigated the expression of circRNAs in the sheep pituitary, finding that circRNAs there participated in the regulation of estrus. By contrast, no puberty- or even estrus-associated study has yet been performed that has tried to identify circRNAs in pigs.

Generally, since gilts have an earlier age at first estrus, they may have a longer productive life, thus farrowing multiple litters and giving birth to more piglets (Patterson et al., 2010; Saito et al., 2011). To reveal the relationships between circRNAs and puberty in the pituitary, here we conducted RNA-seq analyses using pituitaries from Landrace × Yorkshire crossbred pigs at three stages: pre-, in-, and post-puberty, to identify circRNAs and then assemble a circRNA-targeted miRNA-gene network. To our best knowledge, this study is the first to investigate the potential regulatory roles of circRNAs during the onset of puberty in gilts, and so it should provide new insight into this key developmental process at the molecular level.

## Materials and Methods

### Ethics Statement

Animal care and experiments were conducted following the Regulations for the Administration of Affairs Concerning Experimental Animals (Ministry of Science and Technology, China; revised in June 2004) and were approved by the Animal Care and Use Committee of the South China Agricultural University, Guangzhou, China (permit number: SCAU#2013-10).

### Preparation of Animals and Samples

Three stages during the onset of puberty were used: pre-, in-, and post-puberty. The onset of puberty was identified by the standing reflex with the back-pressure test and boar contact (Patterson et al., 2002). A total of nine Landrace × Yorkshire crossbred gilts were used: three gilts of 160 days in age without any pubertal signs were selected as pre-puberty gilts (weight = 81.38 ± 2.40 kg); three gilts exhibiting first pubertal signs served as the in-pubertal gilts (weight = 110.00 ± 2.00 kg); three gilts 14 days beyond the pubertal phase were designated as the post-pubertal gilts (weight = 122.82 ± 9.11 kg). After euthanizing the gilts, their brains were removed immediately and excess tissues were removed. The anterior pituitaries were carefully dissected and frozen immediately in liquid nitrogen, then stored at –80°C until further use.

### RNA Sequencing and Quality Control, and the Transcriptome Assembly

Pre-, in-, and post-pubertal gilts' pituitaries were homogenized separately in liquid nitrogen. The total RNAs were extracted from porcine pituitaries with the Trizol agent (Invitrogen, Carlsbad, CA, USA), followed by quality testing of the total RNAs using the Agilent Bioanalyzer 2100 system (Agilent, Palo Alto, CA, USA). Only those RNA samples with RNA Integrity Number value > 7.0 were deemed eligible. Then, the rRNA from the eligible total RNAs was removed using an Epicentre Ribo-zero rRNA removal kit (Epicentre, Madison, WI, USA). The rRNA-depleted RNAs were used to synthesize double-stranded cDNA *via* the mRNA-Seq Sample Preparation Kit (Illumina, SanDiego, CA, USA), for which a total of 5 μg cDNA per sample was sequenced using a HiSeq 2500 Sequencer according to the manufacturer's instructions, and 150 bp paired-end reads were generated. These raw reads were processed by 3′ adaptor-trimming and removal of low-quality reads—having > 10% unknown bases or > 50% low-mass bases—using Cutadapt software (Martin, 2011). The reads remaining after quality control were defined as the clean reads for further analysis. These acquired clean reads were then mapped onto pig reference genome *Sus scrofa11.1*, using BWA software (Li and Durbin, 2010).

### CircRNA identification

CIRI software (Gao et al., 2015) was applied to obtain the back-spliced junction (BSJ) reads for circRNA prediction based on the annotation file downloaded from the Ensembl genome browser (ftp://ftp.ensembl.org/pub/release-94/gtf/sus_scrofa). Then the number of circRNAs' exons and the length of circRNAs were detected by CIRI-AS module in CIRI software. The expression levels of circRNAs were quantified as the number of reads spanning the BSJ reads in terms of RPM (i.e., mapped BSJ reads per million mapped reads), by using the EBSeq package (Leng et al., 2013). The differential expression of circRNAs was determined according to these criteria: false discovery rate (FDR) < 0.05, log_2_|fold_change| ≥ 1, and circRNA junction reads ≥ 5. Further graphical representations of results were performed in the R platform (R Foundation for Statistical Computing, 2018). Stage-specific circRNAs were defined here as those circRNAs only expressed in one pubertal stage. Known circRNAs of pig were downloaded from the circAtlas 2.0 datasets (Ji et al., 2019), an integrated resource of circRNAs in vertebrates (http://circatlas.biols.ac.cn/). In the circAtlas 2.0 database are tens of thousands of known circRNAs identified from nine porcine tissue types: brain, heart, kidney, liver, lung, skeletal muscle, spleen, testis, and retina. The circRNAs identified in the current study were matched with the database *via* both starting and ending genomic positions of circRNAs, and the novel circRNAs were regarded as the putative tissue-specific circRNAs. Significant differences between any two pubertal pig groups were tested with the Welch two-sample t-test.

### Functional Enrichment Analysis

The circRNAs–miRNAs interactions were predicted with miRanda software (John et al., 2004). These were filtered for predictions with a maximum binding-free energy of less than –20 kcal/mol and a miRanda match score ≥ 150. Next, targeted mRNAs of each selected miRNA were predicted by Targetscan software (Witkos et al., 2011). The competing endogenous RNAs networks among the circRNAs, miRNAs, and mRNAs were built and visualized with Cytoscape software (Su et al., 2014). Functional enrichment analysis was performed using the DAVID bioinformatics resource (Huang et al., 2007). Finally, the Kyoto Encyclopedia of Genes and Genomes (KEGG) pathway and Gene Ontology (GO) terms with Benjamini-Hochberg method-adjusted *P* < 0.05 were identified.

### qRT-PCR Analysis

Quantitative real-time reverse transcription-PCR (qRT-PCR) was carried out using the PrimeScript RT Reagent Kit (TaKaRa, Osaka, Japan) in a Mx3005P real-time PCR System (Stratagene, La Jolla, CA, USA) with SYBR Green, according to the manufacturer's protocol. Divergent primers of 5 circRNAs were designed to further test the accuracy of the RNA-seq, namely Circ 1:14408861|14457143, Circ 9:28120503|28122017, Circ 2:88184110|88206327, Circ 9:75284452|75290025, and Circ 15:74631515|74643464. *GAPDH* served as an internal reference to normalize the expression of circRNAs (**Table S1**). The PCR conditions were 94 °C denaturation for 5 min, 40 cycles at 94 °C for 10 s, 52 to 62 °C for 15 s, and 72 °C for 30 s. The 2^-∆∆Ct^ method was used to analyze the qRT-PCR results. The Student's *t* test was used to assess differences in means of any two pubertal pig groups, for which a *P* < 0.05 was considered statistically significant.

## Results

### Identification of Pituitary-Derived circRNAs During the Onset of Puberty

**Figure 1:**
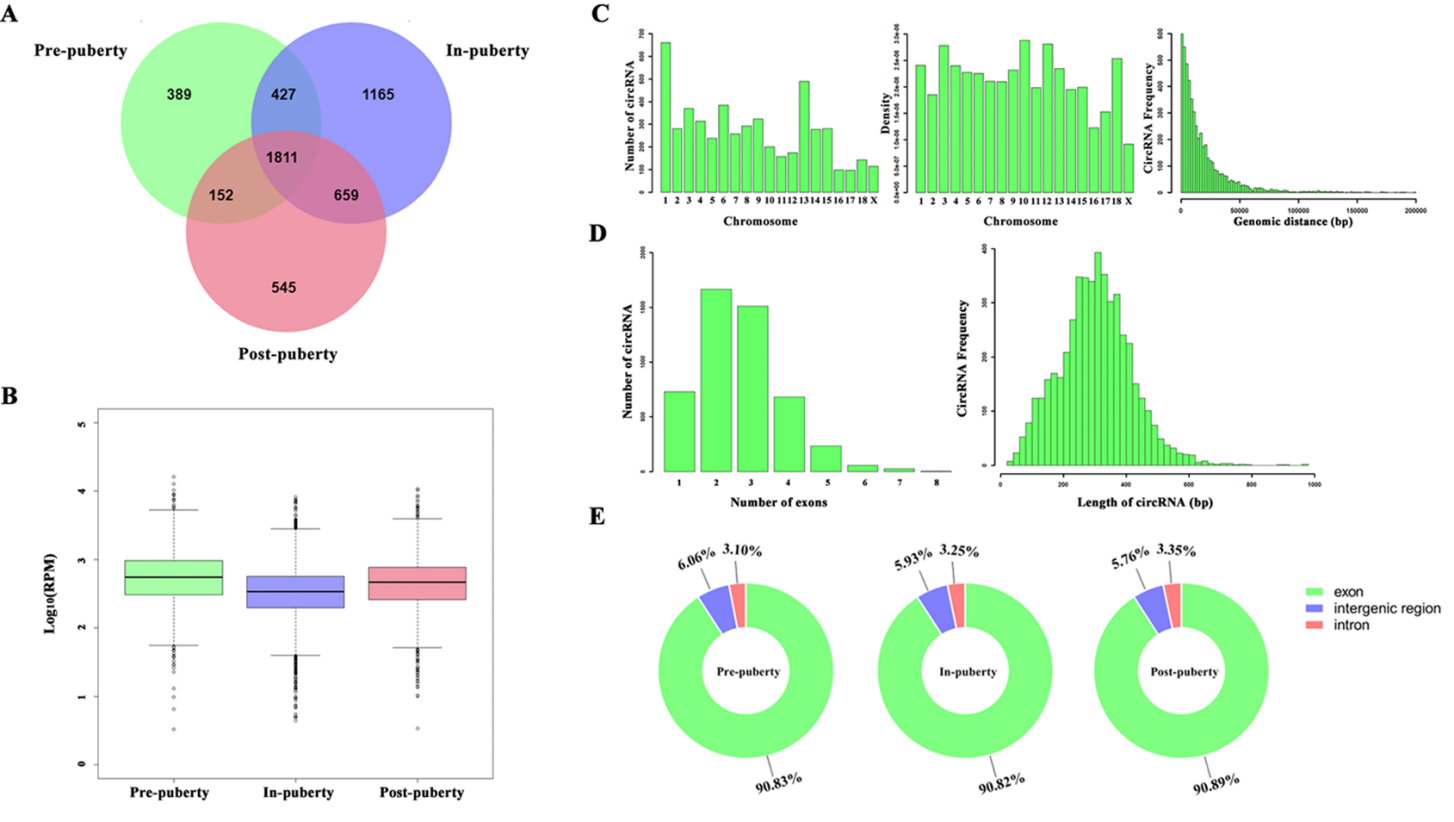
Identification of pituitary-derived circRNAs during the onset of puberty in pigs. **(A)** The Venn diagram of circRNAs detected in pre-, in-, and postpuberty; **(B)** Distribution and genomic distance of the circRNAs; **(C)** Proportion of circRNAs that originated from the exon, intergenic, and intronic regions; **(D)** The exon number of the circRNAs; **(E)** Distribution and transcript length of the circRNAs.

A total of 5148 circRNAs were detected in all three pubertal stages: 2779, 4062, and 3167 circRNAs respectively in the pre-, in-, and post-puberty stages of pigs (**Figure 1A**). The average expression level of circRNAs were dynamically changing during the onset of puberty (**Figure 1B**). *Sus scrofa* chromosome (SSC) 1 harbored the most circRNAs, while the SSC10 had the highest density of circRNAs (**Figure 1C**). The average genomic distance of all circRNAs found was 18 682 bp, with 92% of the circRNAs shorter than 50 000 bp, and the number of circRNAs decreased as their size lengthened (**Figure 1C**). The most circRNAs were made up of two exons, and the length of most circRNAs was about 200 to 300 bp (**Figure 1D**). After annotation with the pig genome, the found circRNAs consisted of approximately 91% exonic, 6% intergenic, and 3% intronic circRNAs, respectively (**Figure 1E**).

### Putative Stage-Specific circRNAs in Gilts During the Onset of Puberty

**Figure 2:**
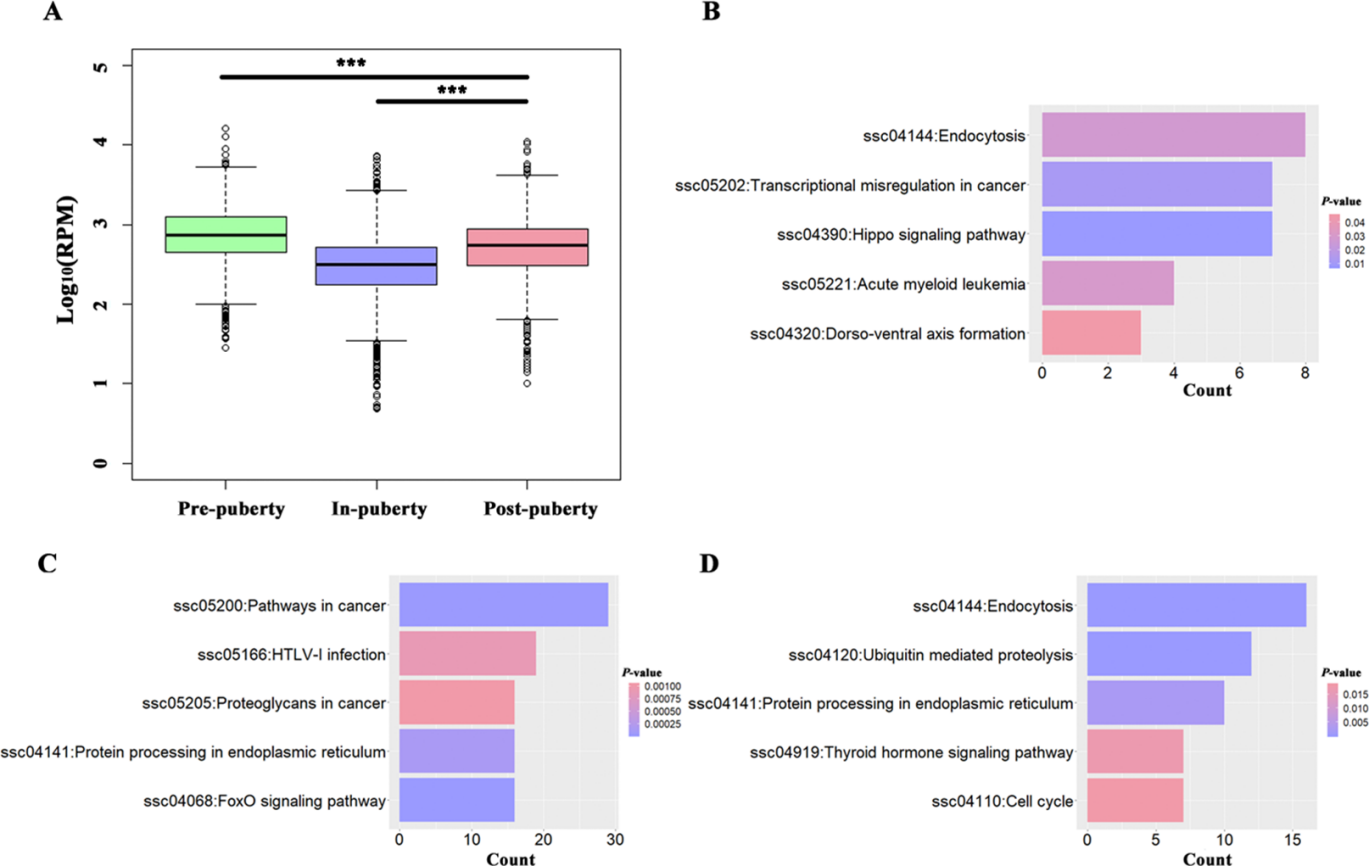
Analysis of potential stage-specific circRNAs in pigs. **(A)** Boxplots of pre-, in-, and post-puberty stage-specific circRNAs**'** expression levels; the top 5 KEGG pathways enriched using parental genes of pre- **(B)**, in-, **(C)** and post-puberty **(D)** stage-specific circRNAs. *** *P* < 0.001.

A total of 389, 1165, and 545 circRNAs were identified as putative stage-specific circRNAs from the pre-, in-, and post-puberty groups, respectively (**Figure 1A**), and their pair-wise comparisons did not reveal any significant difference in bp length (t-test, *P* > 0.05). Further, the expression levels of pre-puberty specific circRNAs significantly exceeded those of post-puberty specific circRNAs (t-test, *P* < 2.20E−16), with the latter being significantly higher than the expression levels of in-puberty specific circRNAs (t-test, *P* < 2.20E−16) (**Figure 2A**). The KEGG pathways enriched using the parental genes of stage-specific circRNAs are listed in **Table S2**, of which the top five are shown in **Figure 2B–D**.

### Putative Tissue-Specific circRNAs in Gilts' Pituitary

**Figure 3:**
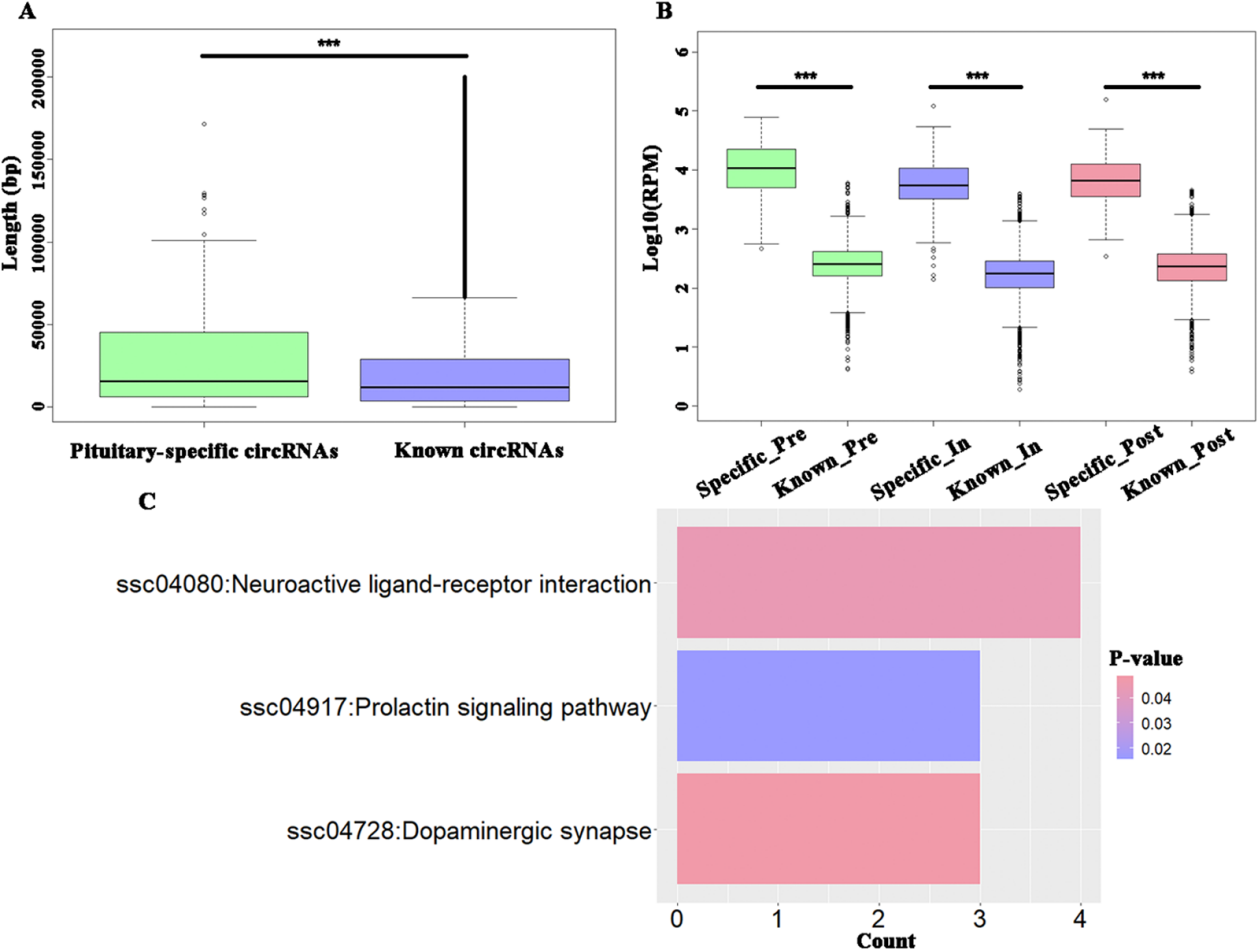
Analysis of potential tissue-specific circRNAs in pigs. **(A)** Boxplots of potential pituitary-specific and known circRNAs**'** length; **(B)** Boxplots of potential pituitary-specific and known circRNAs**'** expression level; **(C)** The KEGG pathways enriched using parental genes of potential pituitary-specific circRNAs. *** *P* < 0.001.

To explore the specific circRNAs in pituitary tissue, 4990 circRNAs were identified as known circRNAs that overlapped with those in circAtlas 2.0, while another 158 circRNAs were identified as being specifically expressed in pituitary tissue. Furthermore, the latter, hereon the “putative pituitary-specific circRNAs,” were significantly shorter than the known circRNAs (t-test, *P* = 7.86E-06) (**Figure 3A**) and these novel circRNAs had significantly higher expression levels than did the known circRNAs during the onset of puberty (t-test, *P* < 2.20E−16) (**Figure 3B**). The KEGG enrichment analysis of parental genes of these putative pituitary-specific circRNAs were enriched in “ssc04917: Prolactin signaling pathway,” “ssc04080: Neuroactive ligand-receptor interaction,” and “ssc04728: Dopaminergic synapse” pathways (**Figure 3C**).

### Analysis of Differentially Expressed circRNAs

A total of 14 differentially upregulated circRNAs and three differentially downregulated circRNAs were identified (**Table 1**). Some of them were derived from different transcripts of the same genes, such as *ESR1* and *RALGPS1*. All differentially regulated circRNAs in the pre- vs. in-puberty groups were both derived from *ESR1* (**Table 1**). Interestingly, one of them, circRNA “Circ 1:14408861|14457143,” was identified here for the first time. Furthermore, the circRNA “Circ 7:121001608|121012600” was downregulated in the in- vs. post-puberty groups yet upregulated in the pre- vs. post-puberty groups.

**Table 1:** The differentially regulated circRNAs in this study of gilts in three pubertal groups.

CircRNA ID	CircRNA type	Position	Strand	Group	Regulation	Parental gene	Top 5 miRNA targets
1:14416335|14457143	Exon	Chr1:14416335–14457143	−	Pre- vs. In-puberty	UP	ESR1	miR-145-5p, miR-145-3p, miR-214-3p, miR-24-3p, miR-140-5p
1:14373973|14457143	Exon	Chr1:14373973–14457143	−	Pre- vs. In-puberty	UP	ESR1	miR-122-5p, miR-145-5p, miR-145-3p, miR-148a-5p, miR-214-3p
1:14408861|14457143	Intron	Chr1:14408861–14457143	−	Pre- vs. In-puberty	UP	ESR1	miR-145-3p, miR-181-5p, miR-26-5p, miR-214-3p, miR-186-3p
2:88184110|88206327	Exon	Chr2:88184110–88206327	−	In- vs. Post-puberty	UP	HOMER1	miR-148a-5p, miR-20a-3p, miR-24-3p, miR-140-5p, miR-214-3p
9:75284452|75290025	Intergenic region	Chr9:75284452–75290025	+	In- vs. Post-puberty	DOWN	—	miR-186-5p, miR-30c-5p, miR-30b-5p, miR-92b-3p, miR-142-5p
7:121001608|121012600	Exon	Chr7:121001608–121012600	+	In- vs. Post-puberty	DOWN	EVL	miR-145-5p, miR-148a-5p, miR-17-5p, miR-30b-3p, miR-221-5p
13:65374802|65376965	Intron	Chr13:65374802–65376965	−	Pre- vs. Post-puberty	UP	SRGAP3	miR-186-5p, miR-199b-5p, miR-331-3p, miR-1306-3p, miR-676-5p
1:264455217|264509476	Exon	Chr1:264455217–264509476	−	Pre- vs. Post-puberty	UP	DENND1A	miR-122-5p, miR-145-3p, miR-148a-5p, miR-148a-3p, miR-24-3p
7:37922045|37927684	Exon	Chr7:37922045–37927684	+	Pre- vs. Post-puberty	UP	BICRAL	miR-145-5p, miR-28-5p, miR-193a-3p, miR-92b-3p, miR-542-3p
3:38413987|38450234	Exon	Chr3:38413987–38450234	+	Pre- vs. Post-puberty	UP	CREBBP	miR-96-5p, miR-214-3p, miR-186-5p, miR-30c-5p, miR-17-3p
1:267525106|267528728	Exon	Chr1:267525106–267528728	+	Pre- vs. Post-puberty	UP	RALGPS1	miR-331-3p, miR-381-3p, miR-1296-5p, miR-126-5p, miR-545-3p
1:267525106|267545008	Exon	Chr1:267525106–267545008	+	Pre- vs. Post-puberty	UP	RALGPS1	miR-331-3p, miR-545-3p, miR-493-5p, miR-381-3p, miR-148a-5p
9:28120503|28122017	Exon	Chr9:28120503–28122017	−	Pre- vs. Post-puberty	UP	MAML2	miR-193a-3p, miR-424-5p, miR-202-5p, miR-490-3p, miR-30e-3p
4:2484106|2484734	Intergenic region	Chr4:2484106–2484734	+	Pre- vs. Post-puberty	UP	—	miR-542-5p, miR-423-5p, miR-181d-5p, miR-345-3p, miR-130b-5p
7:121001608|121012600	Exon	Chr7:121001608–121012600	+	Pre- vs. Post-puberty	UP	EVL	miR-145-5p, miR-148a-5p, miR-17-5p, miR-30b-3p, miR-221-5p
9:75284452|75290025	Intergenic region	Chr9:75284452–75290025	+	Pre- vs. Post-puberty	DOWN	—	miR-186-5p, miR-30c-5p, miR-30b-5p, miR-92b-3p, miR-142-5p
3:84476843|84482961	Intergenic region	Chr3:84476843–84482961	+	Pre- vs. Post-puberty	DOWN	—	miR-145-5p, miR-29a-5p, miR-196b-5p, miR-450b-5p, miR-202-5p

### Validation of circRNAs by qRT-PCR

**Figure 4:**
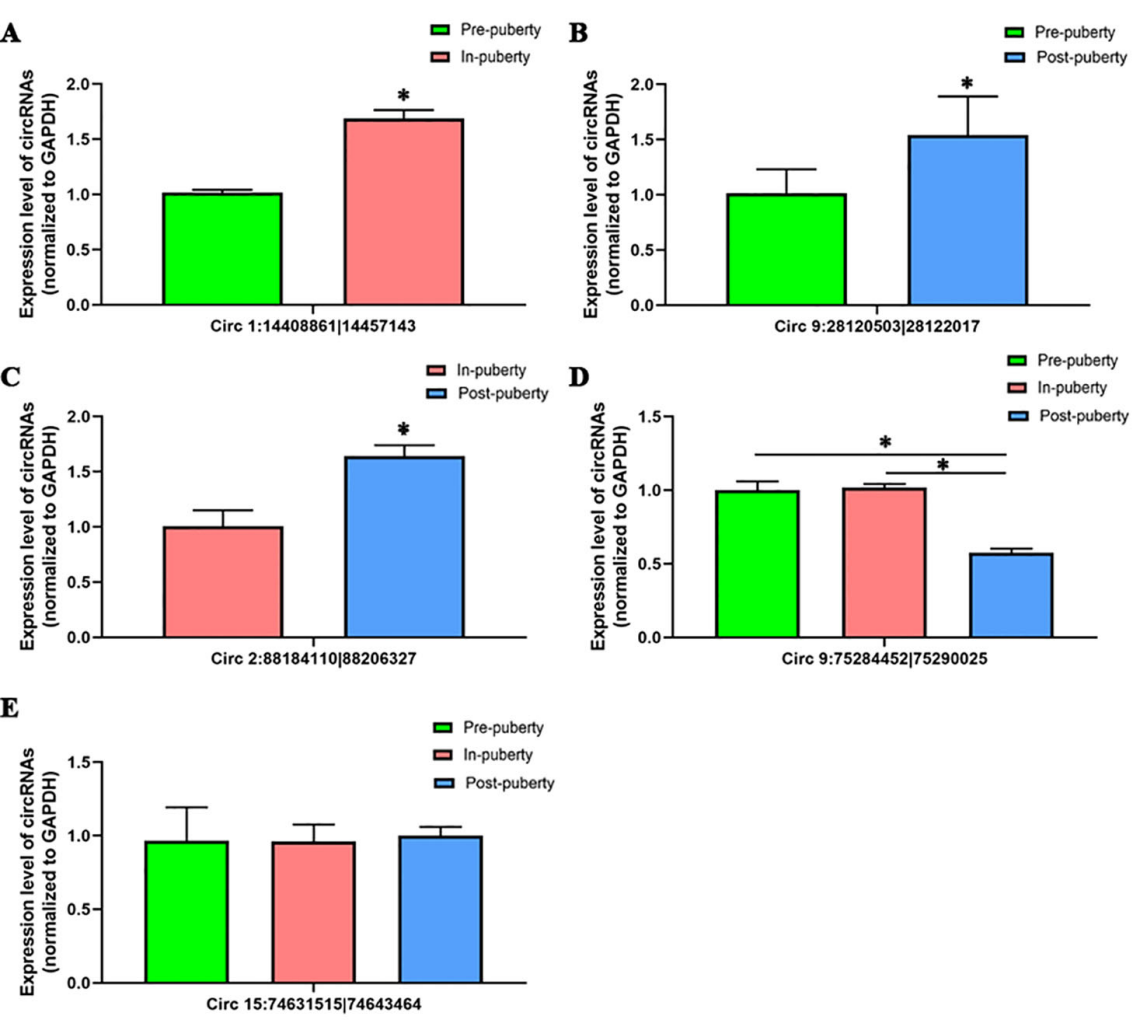
Validation of circRNAs using qRT-PCR. The qRT-PCR results of **(A)** Circ 1:14408861|14457143, **(B)** Circ 9:28120503|28122017, **(C)** Circ 2:88184110|88206327, **(D)** Circ 9:75284452|75290025, and **(E)** Circ 15:74631515|74643464 are shown. The green, red, and blue columns represent the pre-, in-, and post-puberty pig groups, respectively. * *P* < 0.05.

To validate the accuracy of RNA-seq data, a total of five circRNAs, including four differentially expressed circRNAs—Circ 1:14408861|14457143 (**Figure 4A**), Circ 9:28120503|28122017 (**Figure 4B**), Circ 2:88184110|88206327 (**Figure 4C**), Circ 9:75284452|75290025 (**Figure 4D**)—and one randomly selected circRNA: Circ 15:74631515|74643464 (**Figure 4E**) were chosen and validated *via* qRT-PCR.

When compared with the RNA-seq data, similar expression trends for the qRT-PCR results of all selected circRNAs were discovered, thus showing that the obtained qRT-PCR results of these above circRNAs were consistent with the RNA-seq data (**Figure S1**).

### CircRNA-Targeted miRNA-Gene Network Prediction

**Figure 5:**
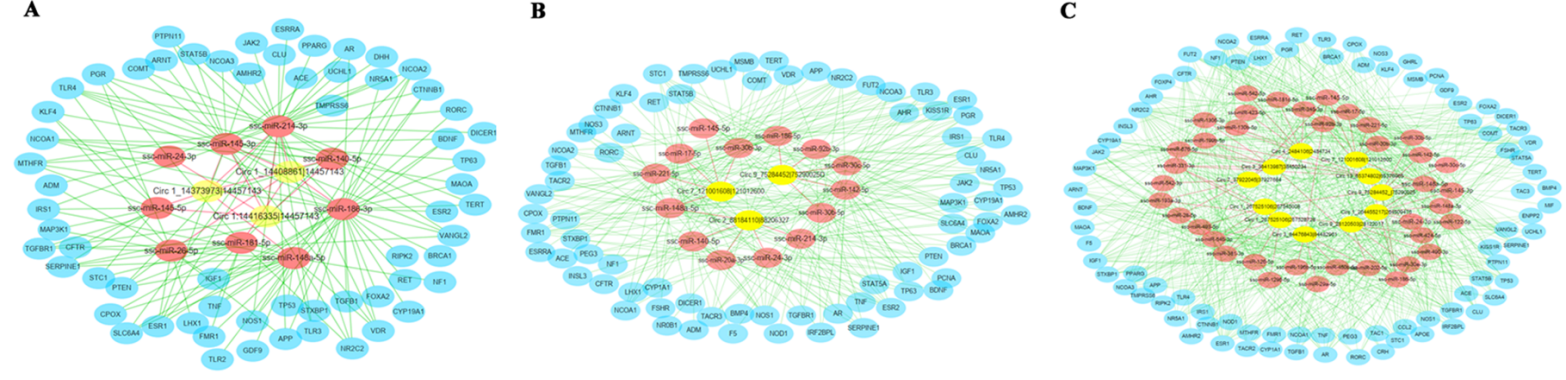
The circRNA-targeted miRNA-gene network prediction results of differentially regulated circRNAs. The network prediction results of differentially regulated circRNAs in **(A)** the pre- vs. in-puberty group, **(B)** the in- vs. post-puberty group, and **(C)** the pre- vs. post-puberty group.

To further explore the putative functions of differentially expressed circRNAs, these circRNAs were conducted to predict the binging sites with miRNA targets (**Figure 5**). The top five plausible miRNA targets were chosen according to their respective miRanda match score and are listed in **Table 1**. According to this study, we found that many of differentially expressed circRNAs interact with miRNAs that potentially regulate estrus of pigs. These predicted circRNA-targeted miRNA-gene networks will be the focus of further research.

## Discussion

As a key physiological process of sexual maturation, the timing of puberty's onset provides a great opportunity for improving the efficiency of gilts' reproduction. In this study, we identified the genome-wide landscape of circRNAs in three important pubertal stages: pre-, in-, and post-puberty. The results showed that the number of circRNAs were dramatically altered among these three stages; the most circRNAs detected from in-puberty pigs, followed by those at post-puberty, with the least number occurring in the stage of pre-puberty. Many genome-wide analyses of circRNAs in mammals have been widely conducted using RNA-seq; these collectively indicate the number of circRNAs can differ between species, as well among different tissues or ontogeny stages. Of the 5148 circRNAs identified in our study, 158 circRNAs were distinguishable as putative pituitary-specific circRNAs that are involved in the prolactin signaling pathway, the neuroactive ligand-receptor interaction, and the dopaminergic synapse. Prolactin secreted by the pituitary was related to the regulation of reproductive function, the immune system, osmotic balance, and angiogenesis (Freeman et al., 2000). The secretion of prolactin is regulated by endocrine neurons between the hypothalamus and pituitary, and its regulation mainly depends on the secretion of dopamine (Bole-Feysot et al., 1998). For the distribution of circRNAs in the genomic regions, previous studies have shown extremely differences between different species and tissues. In pigs, Liang et al. (2017) found that 21.93% of circRNAs in intergenic regions and 68.40% in exon regions through carrying out nine organs. Yan et al. (2018) demonstrated that the found circRNAs consisted of approximately 74.31% exonic, 20.36% intergenic, and 5.33% intronic circRNAs in spleen, and Huang et al. (2018) demonstrated that more than 86% of circRNAs consisted of exons while nearly 10% originated from intronic and intergenic regions in liver. In rats, Yang et al. (2019) found that the circRNAs consisted of approximately 80.18% exonic, 0.15% intergenic, and 19.67% intronic circRNAs in pulmonary. These observations strongly support the view that circRNAs' expression occurs in a specie-specific, tissue-specific, and developmental stage-specific manner.

Importantly, we identified 17 circRNAs that were differentially expressed in the gilts, for which we speculated that some parental genes of differentially regulated circRNAs could influence the fertility and production traits of female mammals, such as *ESR1* (Handa and Weiser, 2014), *DENND1A* (McAllister et al., 2014), *RALGPS1* (Cochran et al., 2013), and *MAML2* (Whittington et al., 2018). In addition, after identifying the miRNA targets of each differentially regulated circRNA, we found that some candidate miRNAs targeted by several circRNAs are linked to mammalian development of sex differentiation and maturation. For example, miR-145-5p was found likewise up-regulated after sexual maturity in pigs (Li et al., 2016) and miR-214-3p was shown to be involved in the onset of mouse primordial germ and somatic cell sex differentiation (Fernández-Pérez et al., 2018). Those findings coupled to our results suggest that circRNAs probably regulate the onset of puberty.

Interestingly, one of the differentially regulated circRNAs, circRNA “Circ 1:14408861|14457143,” was reported here in pig for the first time, and the top five miRNA targets of this particular circRNA had a predicted interaction with *ESR1*. *ESR1* encodes an estrogen receptor alpha, a nuclear receptor activated by the sex hormone estrogen (Green et al., 1986). Previous studies confirmed that lacking an active ESR1 caused the disruption of normal pituitary tissue development and function. For instance, female mice lacking the estrogen receptor alpha in the pituitary gonadotroph have elevated levels of serum LH and LH beta-subunit gene expression, indicating that lacking estrogen has a negative feedback effect on the gonadotroph, with LH values and estrous cyclicity also found absent in these mice (Singh et al., 2009). Most circRNAs detected in our study are in the circAtlas 2.0 database, whose circRNAs were identified by at least two tools (CIRI2, DCC, find_circ, or CIRCexplorer2) to avoid false positives. Hence, the predictions made in the present study should be reliable.

Our dataset provides fresh insight into the existence of pituitary-derived circRNAs in pigs, yet this study did have a few limitations. Although the rRNA-depleted total RNA-seq analyses have been used to enrich for circRNAs in previous studies (e.g., Memczak et al., 2013; Tan et al., 2017; Sekar et al., 2018), there is no doubt that these sequencing analyses may not have comprehensively captured all occurring circRNAs. Furthermore, these enrichment steps may produce a few false BSJ reads that originated from linear RNAs, which could possibly lead to false detections of circRNAs. To guard against this, we used CIRI algorithms to identify circRNAs in this study, as they are reportedly effective for preventing the false detections of circRNAs that are caused by false BSJ reads (Gao and Zhao, 2018). Finally, the underlying mechanism of these circRNAs during pigs' pubertal onset still requires carefully elucidation and verification.

## Conclusions

This investigation identified and described the circRNAs during the onset of puberty in gilts' pituitaries. In all, 5148 circRNAs were found, of which 158 were putative pituitary-specific expressed circRNAs. Because their expression levels were significantly higher than those of the remaining circRNAs during the onset of puberty, this suggested they are involved in regulating the key function of pituitary tissue. Upon further examination, 17 differentially regulated circRNAs were identified and these circRNAs were chosen to construct the posited circRNA-targeted miRNA-gene network. These results suggest circRNAs likely play a critical role in puberty's timing in gilts and thus provide useful information for future investigations of circRNA-mediated puberty at the pituitary level.

## Data Availability Statement

The data used in our study has been released in NCBI Sequence Read Archive with the accession number PRJNA576641 (https://www.ncbi.nlm.nih.gov/bioproject/PRJNA576641).

## Ethics Statement

The animal study was reviewed and approved by The Animal Care and Use Committee of the South China Agricultural University, Guangzhou, China (permit number: SCAU#2013-10).

## Author Contributions

ZC, XP, and YK conceived this study, performed the qRT-PCR validations, and wrote the manuscript. ZZ, YZ, XY, XP, YJ, and JL helped the manuscript review and editing. HZ and ZC originally derived the data and helped in the analyses. JL, ZZ, and XY stimulated the idea of the paper and helped in the manuscript. All authors read, edited, and approved the final manuscript.

## Conflict of Interest

The authors declare that the research was conducted in the absence of any commercial or financial relationships that could be construed as a potential conflict of interest.

## Supplementary Material

Figure S1 Validation of circRNAs using qRT-PCR (left) and comparison withRNA-seq data (right). * P < 0.05.

Table S1 The KEGG pathways of pre-, in-, and post-puberty stage-specific circRNAs.
