# Supplementary figures and images for: Pituitary-Derived Circular RNAs Expression and Regulatory Network Prediction During the Onset of Puberty in Landrace × Yorkshire Crossbred Pigs
Source: Front Genet. 2020 Feb 28;11:135. doi: 10.3389/fgene.2020.00135 (PMC7059797; doi:10.3389/fgene.2020.00135)

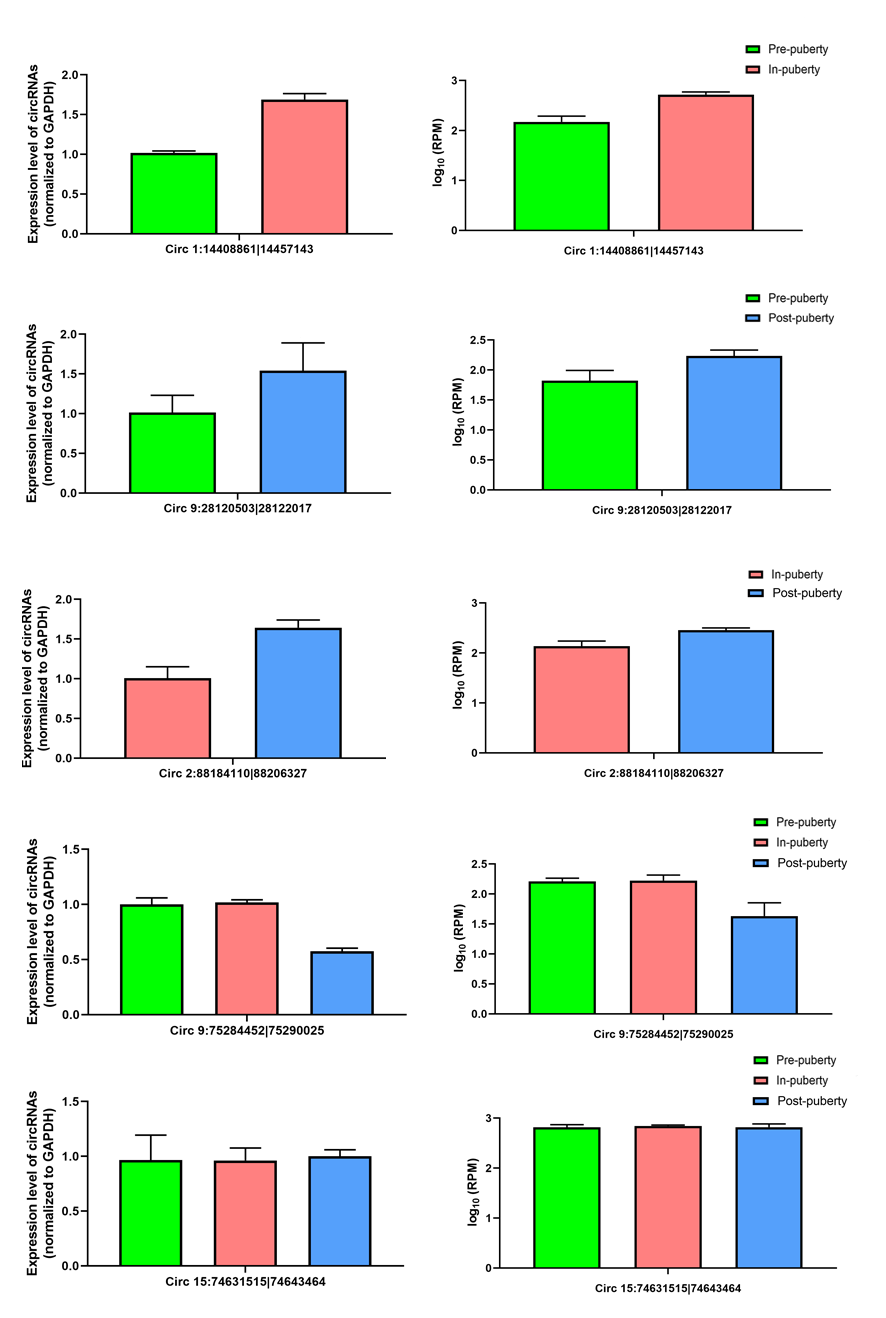

Supplement: Figure S1 — Validation of circRNAs using qRT-PCR (left) and comparison withRNA-seq data (right). * P < 0.05. [file Image_1.tif]
